# Nomograms of Combining Apparent Diffusion Coefficient Value and Radiomics for Preoperative Risk Evaluation in Endometrial Carcinoma

**DOI:** 10.3389/fonc.2021.705456

**Published:** 2021-07-27

**Authors:** Kaiyue Zhang, Yu Zhang, Xin Fang, Mengshi Fang, Bin Shi, Jiangning Dong, Liting Qian

**Affiliations:** ^1^Department of Radiation Oncology, Anhui Provincial Hospital Affiliated to Anhui Medical University, Hefei, China; ^2^Department of Radiology, First Affiliated Hospital of University of Science and Technology of China, Anhui Provincial Cancer Hospital, Hefei, China; ^3^Department of Radiation Oncology, First Affiliated Hospital of University of Science and Technology of China, Hefei, China

**Keywords:** endometrial neoplasms, apparent diffusion coefficient, radiomics, risk stratification, nomogram

## Abstract

**Objectives:**

To evaluate the value of nomogram models combining apparent diffusion coefficient (ADC) value and radiomic features on magnetic resonance imaging (MRI) in predicting the type, grade, deep myometrial invasion (DMI), lymphovascular space invasion (LVSI), and lymph node metastasis (LNM) of endometrial carcinoma (EC) preoperatively.

**Methods:**

This study included 210 EC patients. ADC value was calculated, and radiomic features were measured on T2-weighted images. The univariate and multivariate logistic regressions and cross-validations were performed to reduce valueless features, then radiomics signatures were developed. Nomogram models using ADC combined with radiomic features were developed in the training cohort. The receiver operating characteristic (ROC) curve was performed to estimate the diagnostic efficiency of nomogram models by the area under the curve (AUC) in the training and validation cohorts.

**Results:**

The ADC value was significantly different between each subgroup. Radiomic features were ultimately limited to four features for type, six features for grade, six features for DMI, four features for LVSI, and eight features for LNM for the nomogram models. The AUC of the nomogram model combining ADC value and radiomic features in the training and validation cohorts was 0.851 and 0.867 for type, 0.959 and 0.880 for grade, 0.839 and 0.766 for DMI, 0.816 and 0.746 for LVSI, and 0.910 and 0.897 for LNM.

**Conclusions:**

The nomogram models of ADC value combined with radiomic features were associated with the type, grade, DMI, LVSI, and LNM of EC, and provide an effective, non-invasive method to evaluate preoperative risk stratification for EC.

## Introduction

Endometrial carcinoma (EC) is one of the most common gynecological malignant tumors ranking first among the female genital system in developed countries (1). With the transformation of the social economy, the incidence rate of EC has been rising and the population is becoming younger worldwide because of obesity, low parity, and exogenous estrogen use (2). The primary treatment for EC involves hysterectomy and bilateral salpingo-oophorectomy. The clinicopathological risk factors, including tumor type (type I or type II), histological grade (grade 1, grade 2 or grade 3), FIGO stage, deep myometrial invasion (DMI), lymphovascular space invasion (LVSI), and lymph node metastasis (LNM), guide clinical management and determine the prognosis for EC (3–7). Endometrial biopsy is a crucial method to diagnosing EC, but it has some false-negatives compared with the final surgical pathology. Moreover, the type, grade, myometrial invasion, and lymphatic vascular infiltration cannot be accurately evaluated because of the limitation of materials and heterogeneity of tumor tissue, which may underestimate cancer risk (8, 9). Recently, magnetic resonance imaging (MRI) as a non-invasive preoperative risk assessment method that has been widely used in malignant tumors with functional and molecular imaging technology and reflects both the local and overall characteristics of lesions. The diffusion-weighted imaging (DWI) can identify the histological type of malignant tumor by diffusion of water molecules, which can be quantified by the apparent diffusion coefficient (ADC) value (10, 11). Radiomics can extract features of medical images and transform them into quantitative data, reflecting the intensity, location, and spatial arrangement of voxels, which has been used to evaluate heterogeneity, aggressiveness, and prognosis of malignant tumors (12, 13). The purpose of this study was to develop the nomogram models combining ADC values and radiomics analysis on T2WI to assess the type, grade, DMI, LVSI, and LNM of EC preoperatively.

## Materials And Methods

### Patients

**Figure 1:**
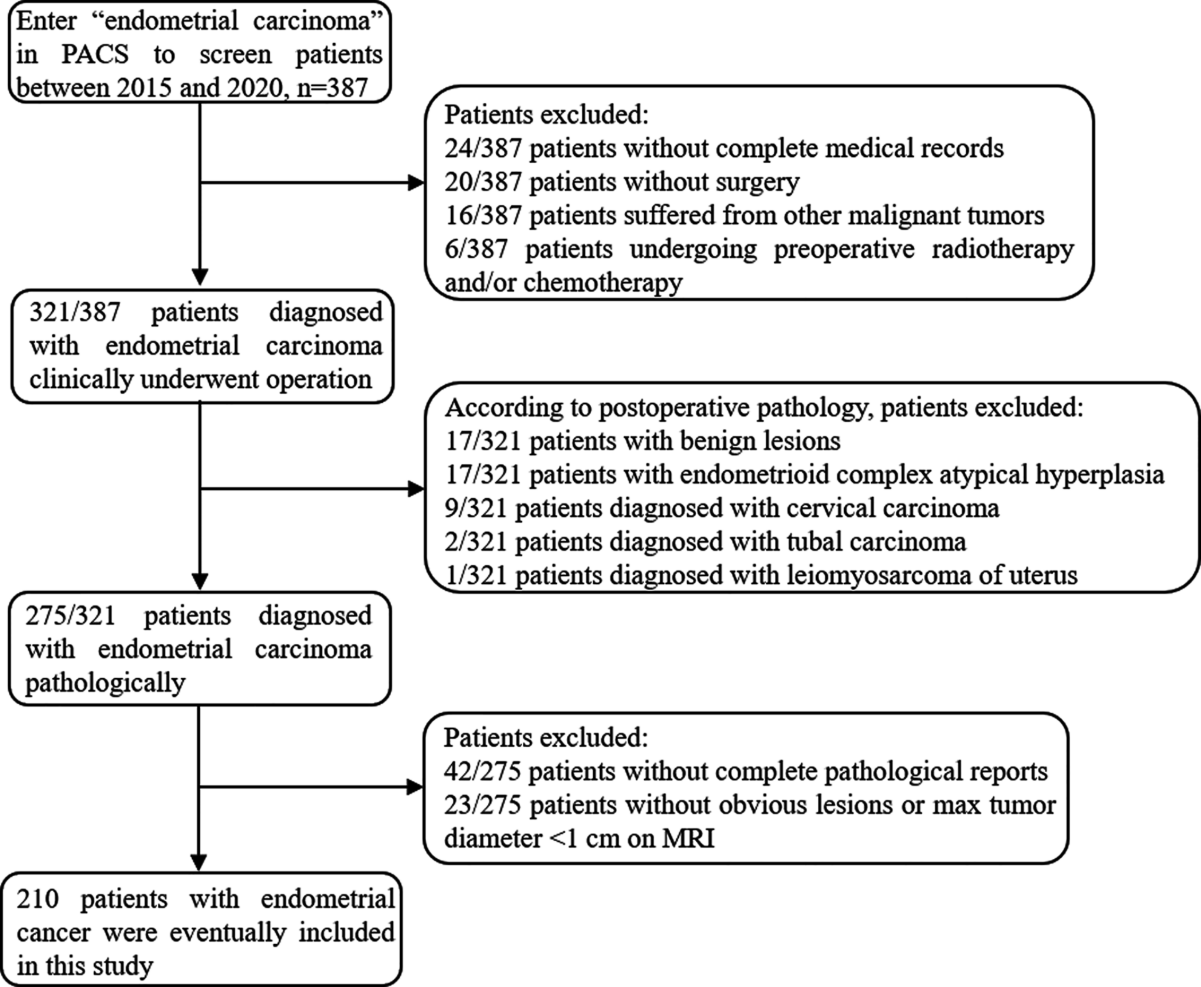
Flowchart shows selection of study population and exclusion criteria.

The institutional review board approved the present retrospective study and the informed consent was waived. Between January 2015 and December 2020, 387 patients who underwent conventional and DWI MRI using a 3T scanner prior to the treatment of EC in our institution were enrolled in the study. The patients were screened through the medical record system of our hospital and were excluded with the following criteria: (a) incomplete medical records or not treated in our institution (n=44), (b) with other malignant tumors (n=16), (c) preoperative chemotherapy or radiotherapy (n=6), (d) non-endometrial carcinoma in postoperative pathology (n=46), (e) incomplete histological diagnosis report (n=42), (f) without obvious lesions or max tumor diameter <1 cm on MRI (n=23). Finally, 210 patients were included in the study (mean age, 56.23 ± 8.60 years; range, 27–88 years). The flowchart of the exclusion process is shown in **Figure 1**. Patients were divided into training cohort and validation cohort at the ratio of 7: 3. This division was carried out in a “stratified sampling” method based on the pathological results to ensure that the frequency of pathological results in training and verification cohort was similar. The clinical and pathological characteristics of patients in the training and validation cohort are described in **Table S1**.

### MR Imaging

All patients underwent conventional pelvic MRI and pelvic axial DWI examination before surgery. MRI was performed using a 3.0-T MR imaging unit system (GE Signa HDXT 3.0T MRI scanner, GE Healthcare, USA) equipped with an eight-channel phased-array body coil. All patients fasted for at least 4 h and were given an intramuscular injection of hyoscine butylbromide 15 mg half an hour before the MRI examination to reduce gastrointestinal peristalsis. The bladder remains semi-filled during the examination to improve the visibility of the lesion without changing the anatomical structure. The patient was supine and breathing calmly during image acquisition, and the scanning range was from aortic bifurcation to the lower margin of the symphysis. Detailed scanning parameters are listed as follows: (1) axial fast spin-echo (FSE) T1-weighted images (T1WI): repetition time (TR)/echo time (TE): 500 ms/7.5 ms, slice thickness: 6 mm, inter-slice gap: 2 mm, a field of view (FOV): 24 cm × 24 cm, and matrix size: 352 × 192. (2) Axial and axial fat suppression (FS) FSE T2-weighted images (T2WI): TR/TE: 4600 ms/68 ms, slice thickness: 4 mm, inter-slice gap: 1 mm, FOV: 24 cm × 24 cm, and matrix size: 320 × 256. (3) Oblique sagittal FSE T2WI: TR/TE: 3220 ms/105 ms, slice thickness: 4 mm, inter-slice gap: 1 mm, FOV: 26 cm × 24 cm, and matrix size: 320 × 256. (4) Axial DWI: TR/TE: 4000 ms/65 ms, slice thickness: 4 mm, inter-slice gap: 1 mm, FOV: 38 cm × 26 cm, and matrix size: 96 × 130, b-value: 0 and 1000 mm^2^/s.

### Image Analysis

**Figure 2:**
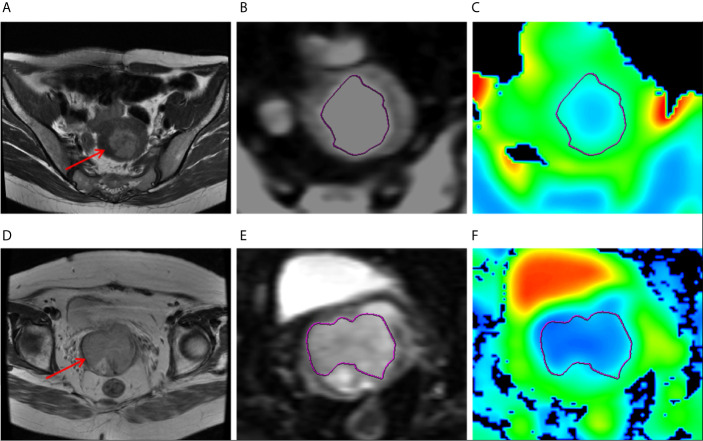
**(A–C)** MR images of a 47-year-old woman with grade 1 endometrioid carcinoma, pathologically proven DMI and absence of LVSI or LNM. **(A)** Axial T2-weighted image showed that there was an irregular mass in the uterine cavity (arrow), the tumor size 3.5 cm × 3.5 cm. **(B)** ROI at the maximum area of the lesion on DWI. **(C)** The ADC map. **(D–F)** MR images of a 47-year-old woman with grade 1 endometrioid carcinoma, pathologically proven DMI, and LVSI but absence of LNM. **(D)** Axial T2-weighted image showed that there was an irregular mass in the uterine cavity (arrow), the tumor size 3.5 cm × 4 cm. **(E)** ROI at the maximum area of the lesion on DWI. **(F)** The ADC map.

Measurement of ADC values: Two subspecialty radiologists with 10 and 12 years of experience in gynecological radiology (reader 1 and reader 2) reviewed the images independently to obtain the ADC values. The original DWI data were imported into GE ADW 4.6 workstation and were analyzed using Function Tool software (GE Healthcare). The function operation based on natural logarithm was carried out on each pixel in DWI (b=0 mm^2^/s and 1000 mm^2^/s) images, that is, the ADC value is calculated by using the following formula: ADC = ln (S0/S1000)/(b1000 − b0), where S represented the signal strength at the corresponding b-value. And the pseudo-color image of ADC was obtained by computer processing. The ROI was manually sketched on DWI image with b=1000 mm^2^/s, and the largest tumor layer was selected to include the whole tumor area, avoiding the surrounding blood vessels and bleeding and necrosis areas. The final ADC value was the average value of the corresponding ROI on the ADC map. The measurement was repeated three times to take the average. The ADC value extraction process is shown in **Figure 2**.

**Figure 3:**
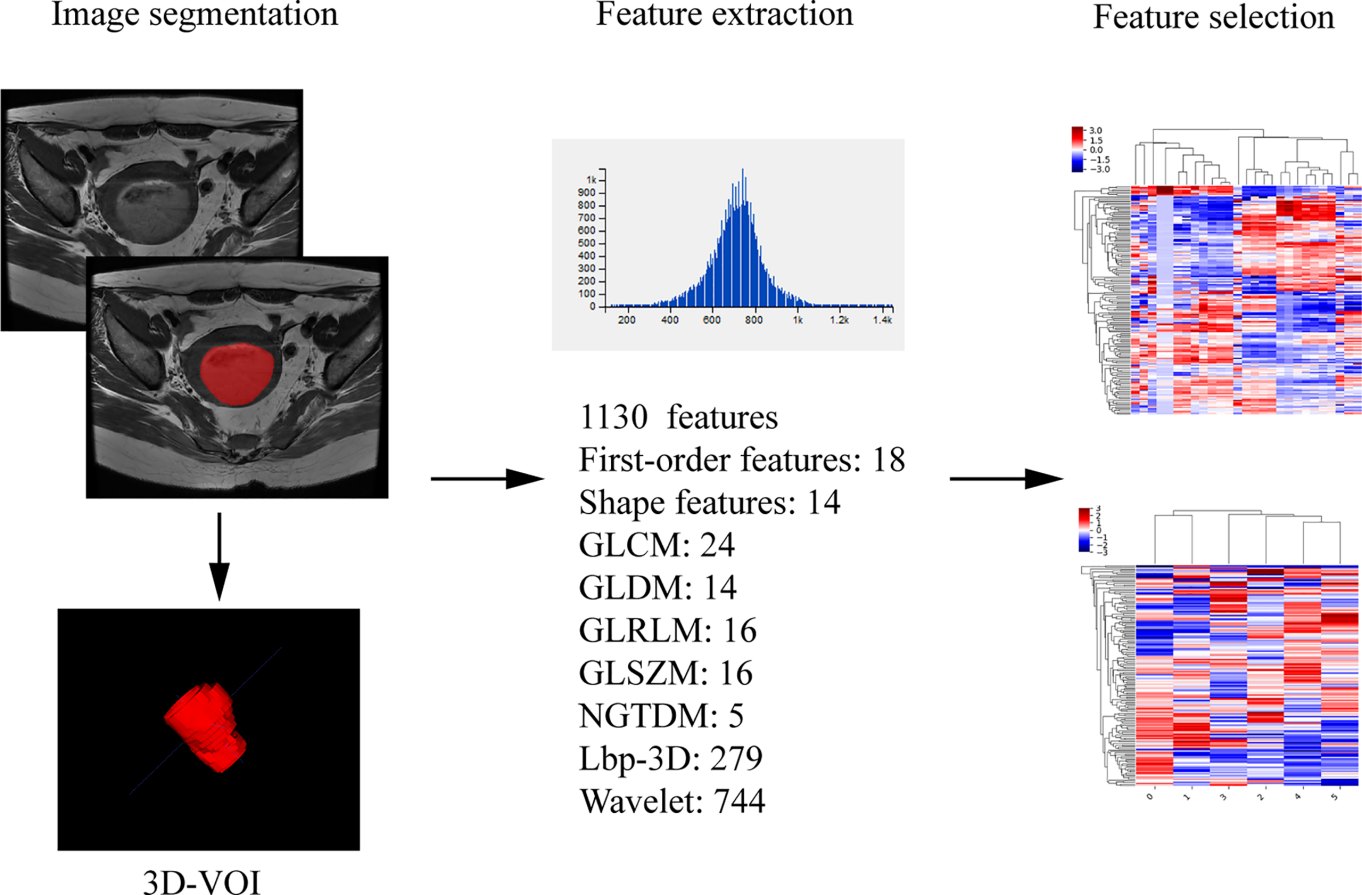
Radiomics workflow.

Assessment of radiomic features: The segmentation was performed on axial T2 weighted image by reader 2. The axial T2WI sequence image were imported into ITK-SNAP (Version 3.6.0, http://www.itksnap.org) software, the ROIs were delineated along the edge of the lesion at each level, which should cover the hemorrhage and necrosis site and avoid the normal anatomical structure. The ROIs were fused into a three-dimensional volume of interest (VOI). Then the VOI files were imported into AK (Analysis Kit, Kinetics Version 2.1, GE Healthcare) software to extract radiomic features. Finally, a total of 1130 radiomic features of the whole tumor were automatically extracted, including the first order, shape features, and wavelet features. The extraction process of radiomic parameters is shown in **Figure 3**. One week later, 30 patients were randomly selected for tumor segmentation and radiomics feature extraction by reader 1 and reader 2. The stability of radiomic features of intra-observer and inter-observer was assessed in these 30 patients.

Image visual evaluation: MR images were reviewed and interpreted independently by two radiologists (with 8 years of experience in pelvic MR diagnosis) (reader 3 and reader 4) who were blinded to clinical and pathological findings. Each reader evaluated the presence of DMI and LNM in combination with T1-weighted images, T2-weighted images, DWI and ADC values, and enhancement sequences.

### Surgical Histologic Findings

The surgical specimens of all patients were reported by two gynecologic tumor pathologists with more than 10 years of experience. The tumor histologic subtype (type I, grade 1 or 2 endometrioid adenocarcinoma; type II, grade 3 endometrioid adenocarcinoma and non-endometrioid subtypes), the histologic grade of endometrioid adenocarcinoma (grade 1, grade 2, grade 3), DMI (the depth of tumor invading muscle layer ≥ 50%), LVSI, and metastases in sampled lymph nodes were confirmed by microscope. Any disagreement was negotiated by the two doctors until an agreement was reached. Distant metastasis was determined by CT or PET-CT, or MRI examination. All patients were staged according to postoperative pathology using the 2018 FIGO staging.

### Statistical Analysis

The data were analyzed by the SPSS 26.0 (IBM Corporation), IPMS (Version 2.4.0, GE Healthcare) and R software (Version 3.6.1, http://www.r-project.org) software. The continuous variables with a normal distribution were presented as mean ± standard deviation with 95% confidence interval (CI), and an abnormal distribution was presented in median. The comparison of ADC values and radiomic parameters among each subgroup was performed using an independent sample *t*-test or Mann–Whitney *U* test, features with *p* < 0.05 were obtained. Univariate and multivariate logistic regression analyses and cross-validations were performed on the radiomic features with statistical significance between each subgroup to select the optimal radiomic features. Pearson correlations were calculated to explore the correlation among radiomic features. If two features had correlation coefficient > 0.75, the feature with smaller regression coefficient was removed. The radiomics signature of each group was developed based on the regression coefficient of the selected features using multi-factor linear weighting in training cohorts, and the radiomics score (radscore) of each patient was calculated. The nomogram models based on ADC value and radscore were built in the training cohorts to predict the type, grade, DMI, LVSI, and LNM of EC, and validated in the validation cohorts. The receiver operating characteristic (ROC) curve was drawn to evaluate the performances of the nomogram models both in training and validation cohort. The intra-observer and inter‐observer consistency of ADC value and radiomic parameters was assessed by intraclass correlation coefficients (ICCs) (ICC >0.7 was indicative of almost perfect agreement). The net reclassification index (NRI) was used to analyze the improvement brought by the combined model compared with other single models. The McNemar test was used to compare the prediction performance of DMI and LNM between the final combined model and human visual assessment in the whole cohort. Kappa analysis was performed to assess the interrater agreement between the combined model and each reader. A K value less than 0.4 is considered poor, 0.41 to 0.6 is considered moderate, 0.61 to 0.8 is considered strong, and more than 0.8 is considered nearly identical. *P* < 0.05 was considered as statistically significant.

## Results

### Clinical and Pathological Findings

Patient age, FIGO stage (2018 International Federation of Gynecology and Obstetrics (FIGO) staging system), and tumor characteristics are presented in **Table 1**. The histologic type was endometrioid carcinoma in 162 patients, including 137 grade 1/2 and 25 grade 3, serous carcinoma in 17 patients, clear cell carcinoma in three patients, carcinosarcoma in 14 patients, neuroendocrine carcinoma in 5 patients, mixed cell adenocarcinoma in 7 patients, and undifferentiated carcinoma in 2 patients. Among the 210 patients, 137 patients were type I EC, and 73 patients were type II EC. The postoperative histologic assessment revealed that 53 (25.2%) of 210 patients had DMI, 42 (20.0%) had LVSI, and 15 (7.1%) had LNM.

**Table 1:** Patients clinical and pathological characteristics.

Variables	Data (n=210)
Ages (year)*	56.23 ± 8.60
**Overall FIGO stage**	
IA	117 (55.7%)
IB	24 (11.4%)
II	42 (20.0%)
IIIA	8 (3.8%)
IIIC1	6 (2.9%)
IIIC2	7 (3.3%)
IVA	1 (0.5%)
IVB	5 (2.4%)
**Histological type**	
Endometrioid	162 (77.1%)
Serous	17 (8.1%)
Clear cell	3 (1.4%)
Carcinosarcoma	14 (6.7%)
Neuroendocrine	5 (2.4%)
Mixed	7 (3.3%)
Undifferentiated	2 (1.0%)
**Type**	
Type I	137 (65.2%)
Type II	73 (34.8%)
**Grade**	n=162
G1/G2	137 (84.6%)
G3	25 (15.4%)
**DMI**	
<50%	157 (74.8%)
≥50%	53 (25.2%)
**LVSI**	
–	168 (80.0%)
+	42 (20.0%)
**LNM**	
–	195 (92.9%)
+	15 (7. 1%)

*Data are mean ± standard deviation. FIGO, International Federation of Gynecology and Obstetrics; G1, grade 1; G2, grade2; G3, grade 3; DMI, deep myometrial infiltration; LVSI, lymphovascular space invasion; LNM, lymph node metastasis.

### The ADC Value Related to the Type, Grade, DMI, LVSI, and LNM of EC

The parametric pseudo-color images obtained by post-processing software based on DWI scanning are shown in **Figure 2**. ICCs of intra-observer were 0.860 (95% CI, 0.631−0.930) and 0.908 (95% CI:0.735−0.956) of reader 1 and reader 2, ICC of inter-observer was 0.872 (95% CI, 0.582–0.966), showing good intra-observer consistency, as well as good inter-observer consistency. The ADC values of reader 2 were accepted because of higher intra-observer consistency. The ADC value of each subgroup is presented in **Table 2**. ADC value was significantly lower in the group of type II EC, G3 endometrioid carcinoma, DMI (≥50%), LVSI (+), and LNM (+) than in the group of type I EC, G1/2 endometrioid carcinoma, DMI (<50%), LVSI (−), and LNM (−) (P<0.05). In the training and validation cohort, the areas under the curve (AUC) of logistic regression model of ADC were 0.691 and 0.681 for type, 0.817 and 0.762 for grade, 0.677 and 0.642 for DMI, 0.731 and 0.689 for LVSI, and 0.657 and 0.638 for LNM, respectively.

**Table 2:** The ADC value in relation to clinical and histologic characteristics.

Variables	ADC (×10^−3^mm^2^/s)	*t* value	*P* value
**Type**		5.334	<0.001
Type I	0.915 ± 0.150		
Type II	0.798 ± 0.153		
**Grade**		4.662	<0.001
G1/G2	0.915 ± 0.150		
G3	0.768 ± 0.125		
**DMI**		3.167	0.002
<50%	0.895 ± 0.160		
≥50%	0.815 ± 0.151		
**LVSI**		4.916	<0.001
–	0.901 ± 0.160		
+	0.770 ± 0.118		
**LNM**		2.198	0.029
–	0.881 ± 0.162		
+	0.787 ± 0.110		

ADC, apparent diffusion coefficient; G1, grade 1; G2, grade2; G3, grade 3; DMI, deep myometrial infiltration; LVSI, lymphovascular space invasion; LNM, lymph node metastasis.

### Radiomics Analysis Related to the Type, Grade, DMI, LVSI, and LNM of EC

A total of 1130 radiomic features were obtained from AK software based on the T2WI sequence. After sufficient dimension reduction with the use of univariate and multivariate analyses and cross-validation in training cohorts, the selected radiomic features were five features for type, six features for grade, six features for DMI, four features for LVSI, and eight features for LNM. The detailed information on these radiomic features is listed in **Table S2**. The intra-observer and inter‐observer ICCs of these parameters selected by the training group were > 0.7 (ICC analysis of radiomic parameters is shown in **Table S3**), showing good intra-observer and inter‐observer consistency. The correlation coefficient maps of selected radiomic features were drawn and presented in **Figure S1**. Finally, the parameter “wavelet-LLL_firstorder_10Percentile” was removed from the model of type. Finally, four radiomic features related to type, six related to grade, six related to DMI, four related to LVSI, and eight related to LNM were obtained. In each group, the radscore of each patient was calculated according to the linear combination of the corresponding regression coefficients in training cohort. The AUC, sensitivity, specificity, and accuracy of the radiomic model for type, grade, DMI, LVSI, and LNM in training and validation cohort are listed in **Table 3**.

**Table 3:** The predictive performance of the models in each group.

Models	Training cohort							Validation cohort						
	AUC (95%CI)	ACC (%)	SEN (%)	SPE(%)	F score (%)	Precision (%)	Recall (%)	AUC (95%CI)	ACC(%)	SEN(%)	SPE(%)	F score (%)	Precision (%)	Recall (%)
**Type**														
ADC model	0.691 (0.590-0.783)	61.9	35.7	88.1	48.4	75.0	35.7	0.681 (0.615-0.741)	62.6	32.6	92.6	46.6	81.6	32.6
Radiomic model	0.839 (0.971-0.884)	75.8	65.3	82.3	72.9	82.7	65.3	0.867 (0.792-0.930)	76.2	76.2	76.2	76.2	76.2	76.2
Combined model	0.851 (0.804-0.895)	80.0	75.8	84.2	79.1	82.8	75.8	0.867 (0.798-0.931)	81.0	85.7	76.2	81.8	78.3	85.7
**Grade**														
ADC model	0.817 (0.768-0.863)	73.2	78.9	67.4	74.6	70.8	78.9	0.762 (0.667-0.841)	69.0	81.0	57.1	72.3	81.0	65.4
Radiomic model	0.917 (0.884-0.948)	86.8	92.6	91.1	87.6	83.0	92.6	0.870 (0.800-0.932)	82.1	81.0	83.3	81.9	82.9	81.0
Combined model	0.959 (0.936-0.978)	90.5	91.6	89.5	90.6	89.7	91.6	0.880 (0.815-0.938)	82.1	83.3	81.0	82.4	81.4	83.3
**DMI**														
ADC model	0.677 (0.616-0.736)	64.2	90.8	37.6	71.7	59.3	90.8	0.642 (0.548-0.732)	64.6	95.8	33.3	73.0	59.0	95.8
Radiomic model	0.828 (0.778-0.875)	77.1	65.0	89.0	74.0	85.5	65.1	0.751 (0.662-0.834)	67.7	54.2	81.2	62.7	74.3	54.2
Combined model	0.839 (0.792-0.883)	78.4	75.2	81.7	77.7	80.4	75.2	0.766 (0.685-0.847)	65.6	52.1	79.2	60.2	71.4	52.1
**LVSI**														
ADC model	0.731 (0.677-0.783)	67.5	98.3	36.8	75.2	60.8	98.3	0.689 (0.605-0.777)	66.7	98.0	35.3	74.6	60.2	98.0
Radiomic model	0.774 (0.722-0.824)	75.2	71.8	78.6	74.3	77.1	71.8	0.668 (0.577-0.757)	65.7	58.8	72.5	63.2	68.2	58.8
Combined model	0.816 (0.769-0.86)	75.2	76.1	74.4	75.4	74.8	76.1	0.746 (0.662-0.827)	68.6	62.7	74.5	66.7	71.1	62.7
**LNM**														
ADC model	0.657 (0.572-0.743)	66.1	98.3	33.9	74.8	60.0	99.3	0.638 (0.58-0.692)	66.5	99.3	33.8	74.4	59.8	98.3
Radiomic model	0.901 (0.867-0.933)	85.7	89.0	82.4	86.1	83.4	89.0	0.895 (0.844-0.942)	81.4	91.5	71.2	83.1	76.1	91.5
Combined model	0.910 (0.878-0.942)	84.9	97.8	72.1	86.6	77.8	97.8	0.897 (0.841-0.944)	81.4	93.2	69.5	83.3	75.3	93.2

AUC, area under the curve; CI, confidence interval; ACC, accuracy; SEN, sensitivity; SPE, specificity; ADC, apparent diffusion coefficient; DMI, deep myometrial infiltration; LVSI, lymphovascular space invasion; LNM, lymph node metastasis.

### Diagnostic Performance of Nomogram Based on ADC Value Combined With Radiomics

**Figure 4:**
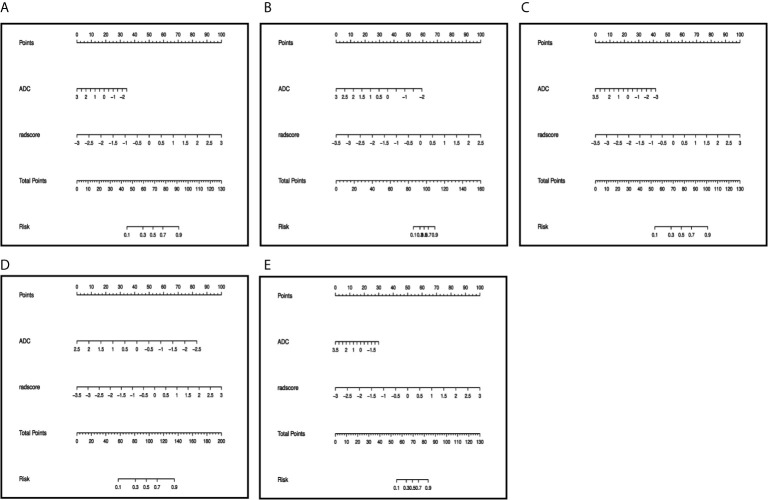
Preoperative nomogram of the combined model for **(A)** type, **(B)** grade, **(C)** DMI, **(D)** LVSI, **(E)** LNM. The different value for ADC and radscore corresponds to a point on the line, total point is calculated by adding all the points up. And the final predicted value was corresponded to the total point.

**Figure 5:**
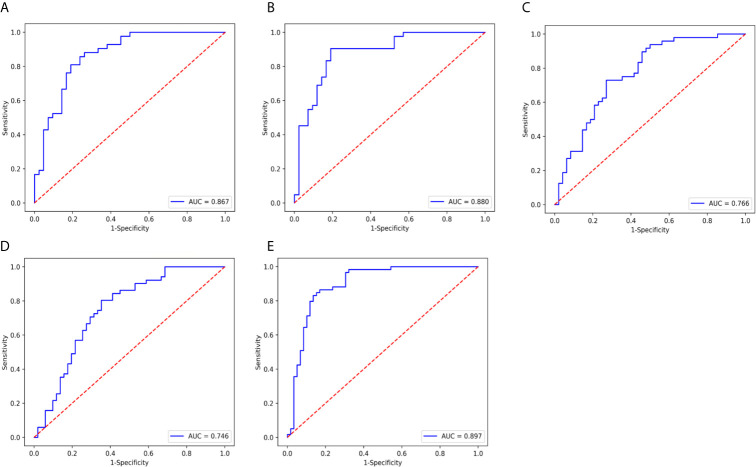
ROC curve of the nomogram model in validation cohort. **(A)** type, AUC=0.867. **(B)** grade, AUC= 0.880, **(C)** DMI, AUC=0.766. **(D)** LVSI, AUC= 0.746. and **(E)** LNM, AUC=0.897.

**Figure 6:**
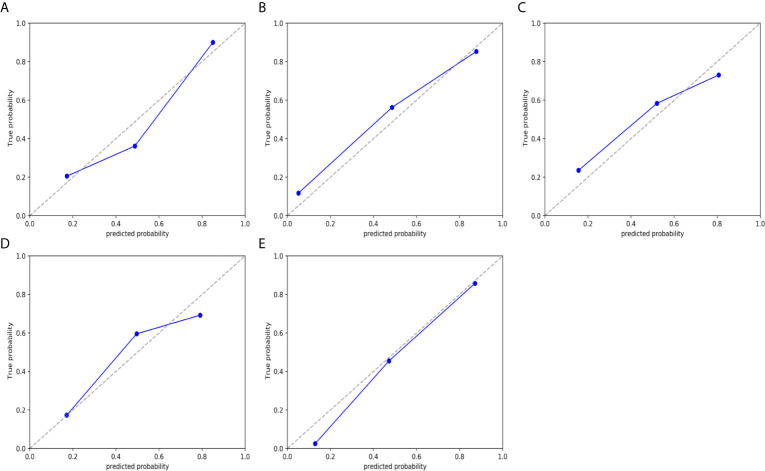
Calibration curves of the nomogram model in validation cohort. **(A)** type. **(B)** grade. **(C)** DMI. **(D)** LVSI. and **(E)** LNM. The 45° dotted line represents the ideal prediction, while the blue line represents the prediction performance of the nomogram. The closer the blue line is to the dotted line, the better the performance of the nomogram.

The ADC value and radiomic features were used to develop nomograms in the training cohort (**Figure 4**). The score of ADC value and radiomics score were weighted by the hazard ratio, and the total score is obtained by combining them together. Finally, probability value corresponding to the total score in nomogram was used to evaluate the risk of type II, high grade, DMI, LVSI, and LNM of patients with EC. The AUC, sensitivity, specificity, and accuracy of the nomogram models to predict the type, grade, DMI, LVSI, and LNM in the training and validation cohorts are shown in **Table 3**, and the ROC curves of validation cohorts are presented in **Figure 5**. The calibration curves of each validation group are shown in **Figure 6**, and the statistics of Hosmer–Lemeshow test in each group are not significant (p > 0.05), indicating good calibration. The decision curves of validation cohorts are listed in **Figure S2**, when the threshold probability of patients is within the corresponding range (the threshold probability is between 0 and 0.87 for type, 0.13 and 0.93 for grade, 0 and 0.70 for DMI, 0 and 0.68 for LVSI, and 0 and 0.86 for LNM), the use of nomogram will increase the net benefit.

Based on the cutoff value determined by Youden index, the (NRI) was used to analyze the improvement brought by the combined model compared with other single models in the training and validation cohort (**Table S4**). In general, the diagnostic efficiency of the combined models was better than that of the single ADC and radiomic models (NRI > 0), but in the training group of LVSI and LNM and the validation group of type, the combined models didn’t outperform the single radiomic model.

The sensitivity, specificity, and accuracy values of the combined model and visual assessment for DMI and LNM are shown in **Table S5**. Using the McNemar test, for DMI, the sensitivity and accuracy showed no significant difference between the combined model and the radiologists’ visual reviews, and the specificity was significantly high of combined model than that of the radiologists’ visual reviews. For LNM, the sensitivity, specificity, and accuracy showed no significant difference between the combined model and the radiologists’ visual assessment (**Table S5**). For DMI, the interrater agreement was poor between the combined model and reader 1 and reader 2 (k=0.23; 0.35). The interrater agreement among the two readers was strong (k=0.74). For LNM the interrater agreement was moderate between the combined model and reader 1 and reader 2 (k=0.41; 0.45). The interrater agreement among the 2 readers also was strong (k=0.78).

## Discussion

Our present study developed a series of nomogram models based on ADC value combined with radiomics analysis derived on T2WI to provide a practical clinical tool for preoperatively risk stratification and individualized treatment of EC. Although most EC can be diagnosed at an early stage, but the difference in patient characterizes and histopathological features affect both the choice of treatment methods and prognosis (3). Clinically, EC is divided into a high-risk group and low-risk group according to FIGO stage, type, grade, and biological behavior (including DMI, LVSI, and LNM). For the low-risk group, lymphadenectomy may not be recommended because of the risk for complications without improved prognosis, and fertility-sparing treatment may be available for low-grade type I EC without DMI. For high-risk group, especially with DMI, LVSI, and LNM, lymph node dissection, cytoreductive surgery, chemotherapy, and radiotherapy are necessary to improve the outcome (14, 15). However, accurate risk index results can only be obtained after surgery, which may lead to underestimation of tumor risk or over-treatment. So, it is very important to establish a noninvasive preoperative risk stratification model to remedy the defect of biopsy and guide the treatment of EC.

ADC value can reflect the diffusion of water molecules in different tissue microenvironment and has been shown to correlate inversely with the proliferation degree and cell density of tumor (16). The present study suggested that the ADC value was significantly lower in the group of type II EC and G3 endometrioid carcinoma than in the group of type I EC and G1/2 endometrioid carcinoma, which was consistent with the previous researches (10, 17–19). Chen et al. (17) and Reyes-Pérez et al. (18) reported that ADC value can be used as a marker of tumor proliferation and solid components. The type II EC consists of poorly differentiated subtypes, such as serous carcinoma, carcinosarcoma, and clear cell carcinoma, and the glandular structure is reduced, even disappeared, and replaced by flaky or papillary tumor tissue with a dense arrangement. Similarly, high-grade endometrioid adenocarcinoma has fewer glandular components and more solid components (20). Therefore, lower ADC values are indicative of type II and high-grade EC. However, the research of Bonatti et al. (6) showed that ADC values did not show any statistically significant correlation with tumor grade, and the reason may be related to differences in the study groups. The grade in this study was for endometrioid adenocarcinoma only, while Bonatti’s (6) study included non-endometrioid adenocarcinoma and classified it as grade 3, and the sample size could also affect the results.

In addition, the present study also found that the ADC values of DMI, LVSI, and LNM groups were significantly reduced. As reported in previous studies, DMI, LVSI, and LNM are more likely to occur in type II EC, which has higher heterogeneity and invasiveness (6), indicating that cancer tissue has more solid components and more likely to invade surrounding tissues and metastasize (5). Moreover, the premise of metastasis and invasion is the proliferation and diffusion of tumor parenchymal cells, which will also increase the density of tissue cells and decrease the ADC value. The research of Reyes-Pérez et al. (18) showed that both ADC_min_ and ADC_mean_ values were lower for EC patients with DMI than for those with superficial muscular infiltration. Zhang et al. (21) reported that ADC value was significantly lower in tumors with LVSI compared to those without LVSI, diversely, Lavaud and his colleagues (22) found that there were no significant differences in ADC between presence or absence of LVSI in EC. Regarding the LNM, there were no significant differences in ADC value between EC patients with LNM and those without LNM in the study of Fasmer et al. (23). All of these findings indicate that ADC values are still controversial in identifying the biological behavior of EC. Although our study showed that ADC values differed among different risk factors subgroups, the AUC of ROC curve of ADC value prediction type II EC, high-grade endometrioid adenocarcinoma, DMI, LVSI, and LNM were 0.681, 0.762, 0.642, 0.689 and 0.638, respectively, which had poor performance. This may be because the ADC value may be affected by blood microcirculation and cannot truly reflect the movement of water molecules in tissues. Besides, the measurement methods of each center are different, which will also affect the results. The low diagnostic efficiency of DMI, LVSI, and LNM may be related to the lack of positive samples and the heterogeneity of histological types.

We performed radiomics analysis on T2WI. In the selected parameters, only the “firstorder_10Percentile” of type and LVSI and the “firstorder_Minimum” of grade were first-order features, which described the distribution of voxel intensity within an image region defined by a mask. Others were second-order or high-order features, which described the local features of adjacent pixels or the spatial distribution relationship and probability of pixels, including GLCM, GLSZM, GLDM, wavelet transform features and so on. It can be seen that the second-order and higher-order features can provide more microscopic information of the tumor. Among these radiomic features, “firstorder_10Percentile” was related to both type and LVSI, “wavelet-HLH_firstorder_Skewness” was related to both type and grade, and the rest of the features were different. We calculated the radscore based on a linear combination of the regression coefficients of each parameter, which integrated the effects of each parameter.

In the past few years, several studies have shown that radiomics analysis can provide effective information for the diagnosis, prognosis, and treatment response assessment of tumors, although the methods vary (24–26). Xu et al. (24) combined radiomics, CA125, and lymph node size to develop a model for predicting LNM of EC, which showed a good discriminant ability. Ueno et al. (25) established random forest models with texture parameters to predict MDI, LVSI, and high-grade endometrial carcinoma, with the AUC of 0.84, 0.80, and 0.83, respectively.

Different from previous studies, we established a series of nomogram models that integrating the ADC value and radiomics and covered five risk factors of EC. When ROC was used to evaluate the performance of the prediction model, the AUC performs well with the range of 0.7 to 0.9. Because of the limited sampling of endometrial biopsy, the biopsy reports of a considerable number of patients in our study were only “endometrial cancer” without providing detailed classification and grading, so the comparison between the model and the biopsy results was not conducted in this study. However, we found that many previous studies had investigated the correlation between the type and grade of tumor in preoperative specimens of EC women and the final specimens. The study of Batista et al. (27) showed that the overall concordance of preoperative histological examination for final surgical pathology grading in EC was 60.75% to 67.24% for G1, 43.75% for G2, and 40% for G3 tumors, which indicated that preoperative endometrial sampling was only a modest predictor of postoperative histological grading. Similar results were found in the research of Piotto et al. (28), which showed that the general consistency between preoperative endometrial specimens and surgical specimens was 46.8% (kappa: 0.279, *P <*0.001), and the agreement varied significantly by tumor type and grade (endometrioid adenocarcinoma grade 1, 64.0%; and grade 2, 50.0%; and non-endometrioid adenocarcinoma, 81.8%). A meta-analysis involving 45 studies found that the pooled agreement rate between preoperative endometrial sampling and final diagnosis for tumor grade was 0.67 (95% CI, 0.60–0.75), and Cohen’s k was 0.45 (95% CI 0.34–0.55). Agreement on histologic subtypes was 0.95 (95% CI 0.94–0.97) and 0.81 (95% CI 0.69–0.92) for preoperative endometrioid and non-endometrioid carcinomas (29). Overall, preoperative biopsy has limitations in predicting tumor type and grade compared with the final surgical specimen and should be complemented with other methods to better plan the surgical management strategy. In our study, the nomogram performed well in identifying type and grade of EC, with the AUC of 0.851 and 0.867 for type, 0.959 and 0.880 for grade in training cohort and validation cohort, respectively, and increased the value of preoperative prediction type and grade when compared with preoperative biopsy alone.

This nomogram was established on the basis of EC confirmed by preoperative biopsy. Although it shows good differential ability, it cannot replace the position of endometrial biopsy in the diagnosis of EC. However, the combination of the two may improve the predictive value of EC type and grade and better plan the surgical strategy.

Up to now, many studies have explored the preoperative risk assessment model of EC, although the factors and methods vary. The study of Prueksaritanond et al. (30) suggested that serum human epididymis protein 4 (HE4) was significantly correlated with primary tumor diameter and depth of myometrial invasion, but the predictive value of HE4 on muscular invasion was not further studied. Reijnen et al. (31) developed a Bayesian network model for predicting the LNM of EC preoperatively, and the predictive factors included the following: preoperative grade; immunohistochemical expression of estrogen receptor (ER), progesterone receptor (PR), p53, and L1 cell adhesion molecule (L1CAM); cancer antigen 125 serum level; thrombocyte count; imaging results on lymphadenopathy; and cervical cytology. The AUC of the model in the validation cohort was 0.82 (95% CI, 0.76–0.88). Although the efficacy is well, this model has high requirements for preoperative information, especially histology. In practice, the preoperative immunohistochemistry of a considerable number of patients is not perfect or even missing because of the limited intimal sampling or other reasons, so the clinical application of this model is limited. Luo et al. (26) combined age, grade, and radiology to construct a nomogram for predicting LVSI of EC with the AUC of 0.820 and 0.807 in the training and test cohort. However, they only extracted 107 radiological features, which may not include other features with strong correlation with LVSI. Our study aimed to mine more information about EC risk from MRI (routine preoperative examination of EC) for preoperative risk assessment, and finally established the nomograms combining ADC and radiomics (selected from 1130 radiomic features). Despite containing only MRI-related features, our nomograms have achieved similar predictive value as previous studies, especially in LNM, which may have higher value when combined with clinicopathologic factors for preoperative evaluation of EC.

When using the NRI to evaluate improvements to the combined model, the diagnostic efficiency of the combined models was better than that of the single ADC and radiomic models in general, but in the training group of LVSI and LNM and the validation group of type, the combined models did not outperform the single radiomic model. This shows the limitation of ADC value in improving the diagnostic performance of the model. In addition, there were many histological types of type II, and the positive ratio of LVSI and LNM was small, which also affected the efficiency of the combined model. The efficiency of the combined model may be improved when the sample size is further expanded and the histological types are stratified.

The human eye cannot detect tumor type, grade and the presence of LVSI on MR images, our study provides a new perspective for preoperative evaluation of MRI. For DMI and LNM, the models achieved accuracy comparable to or slightly higher than that of experienced radiologists.

There are several limitations to our study. First, as a retrospective study, the standardization of images may be limited. Second, we employed only the T2WI sequence for radiomics analysis, and some lesion information may be missed. Further study is needed to perform the radiomics analysis on multi-sequence. Third, our model did not include FIGO stage, Ki-67 and other clinicopathological indicators, and lacks external verification, which requires further study of multi-center large samples.

## Data Availability Statement

The raw data supporting the conclusions of this article will be made available by the authors, without undue reservation.

## Ethics Statement

The studies involving human participants were reviewed and approved by Anhui Provincial Hospital Ethics Committee. Written informed consent for participation was not required for this study in accordance with the national legislation and the institutional requirements.

## Author Contributions

LQ, JD, KZ, YZ, BS, and MF: conception and design. KZ, YZ, and XF: collection and arrangement of data. KZ, YZ, MF, BS, LQ, and JD: data analysis and manuscript writing. All authors contributed to the article and approved the submitted version.

## Funding

This work was supported by 2020 SKY Image Research Fund (NO. Z-2014-07-2003-11) and National key research and development program (No. 2016YFB1000905).

## Conflict of Interest

The authors declare that the research was conducted in the absence of any commercial or financial relationships that could be construed as a potential conflict of interest.

## Publisher’s Note

All claims expressed in this article are solely those of the authors and do not necessarily represent those of their affiliated organizations, or those of the publisher, the editors and the reviewers. Any product that may be evaluated in this article, or claim that may be made by its manufacturer, is not guaranteed or endorsed by the publisher.

## Supplementary Material

Supplementary Figure 1 The correlation coefficient figure of selected parameters of **(A)** type. **(B)** grade. **(C)** DMI. **(D)** LVSI. and **(E)** LNM.

Supplementary Figure 2 Decision curves in verification cohort. when the threshold probability of patients is within the corresponding range, the use of nomogram will increase the net benefit. **(A)** type, the threshold probability is between 0 and 0.89. **(B)** grade, the threshold probability is between 0.13 and 0.93. **(C)** DMI, the threshold probability is between 0. and 0.70. **(D)** LVSI, the threshold probability is between 0 and 0.68. and **(E)** LNM, the threshold probability is between 0. and 0.86.
