# Supplementary figures and images for: Nomograms of Combining Apparent Diffusion Coefficient Value and Radiomics for Preoperative Risk Evaluation in Endometrial Carcinoma
Source: Front Oncol. 2021 Jul 27;11:705456. doi: 10.3389/fonc.2021.705456 (PMC8353445; doi:10.3389/fonc.2021.705456)

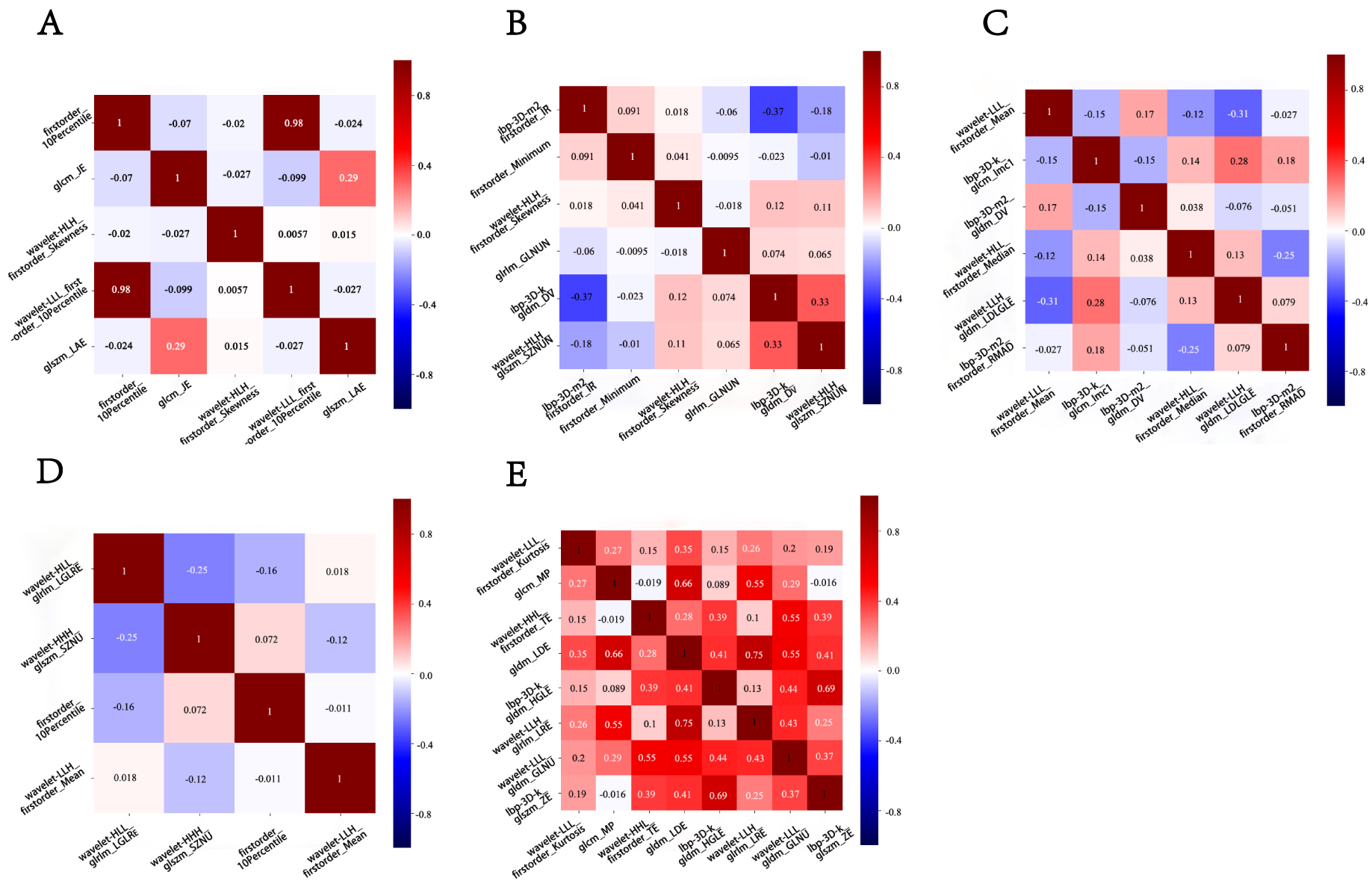

Supplement: Supplementary Figure 1 — The correlation coefficient figure of selected parameters of (A) type. (B) grade. (C) DMI. (D) LVSI. and (E) LNM. [file Image_1.tif]

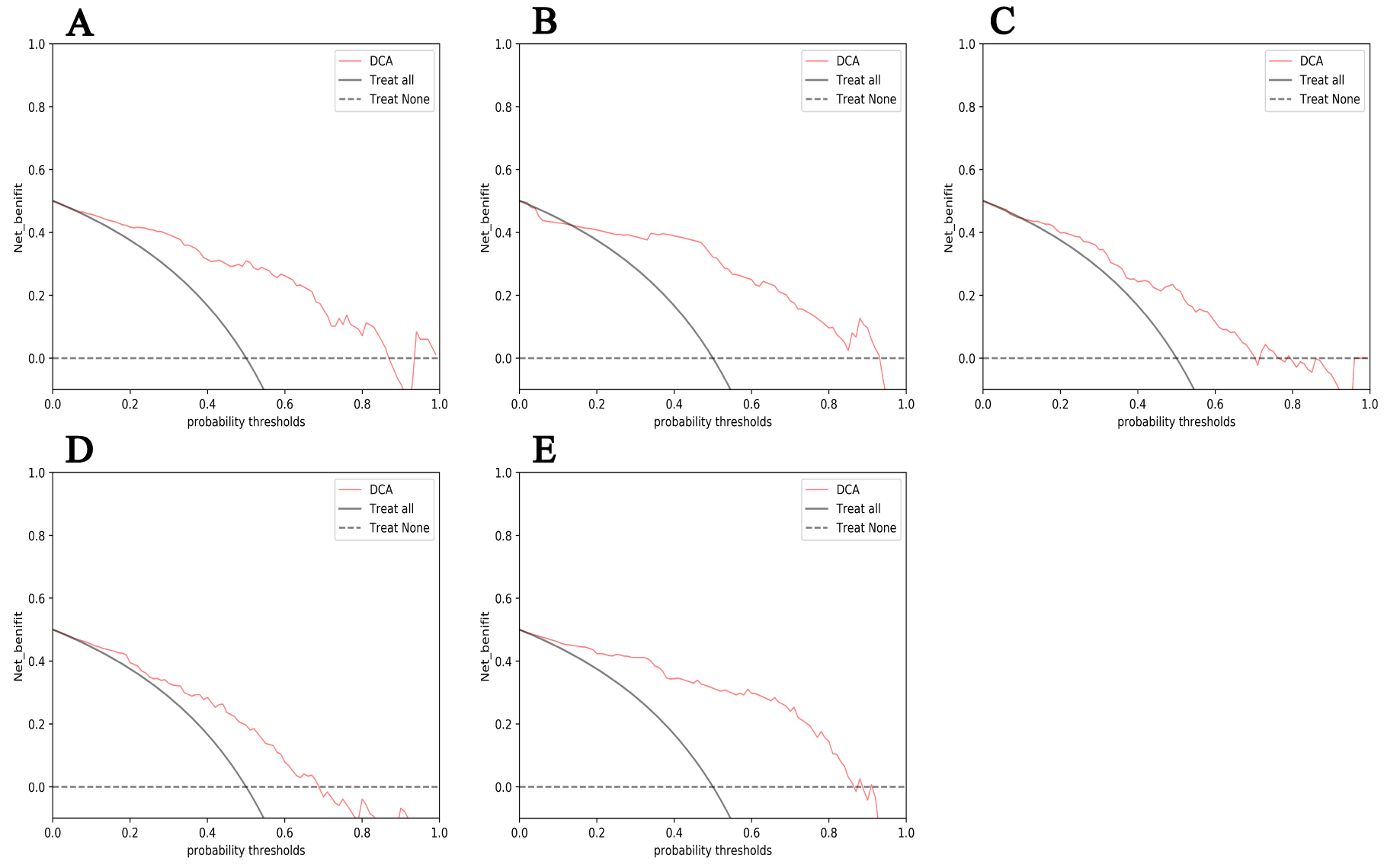

Supplement: Supplementary Figure 2 — Decision curves in verification cohort. when the threshold probability of patients is within the corresponding range, the use of nomogram will increase the net benefit. (A) type, the threshold probability is between 0 and 0.89. (B) grade, the threshold probability is between 0.13 and 0.93. (C) DMI, the threshold probability is between 0. and 0.70. (D) LVSI, the threshold probability is between 0 and 0.68. and (E) LNM, the threshold probability is between 0. and 0.86. [file Image_2.tif]
