# Supplementary material for: Nomograms of Combining Apparent Diffusion Coefficient Value and Radiomics for Preoperative Risk Evaluation in Endometrial Carcinoma
Source: Front Oncol. 2021 Jul 27;11:705456. doi: 10.3389/fonc.2021.705456 (PMC8353445; doi:10.3389/fonc.2021.705456)
Supplement: Supplementary file 3 [file Table_1.docx]

Table S1. Patients clinical and pathological characteristics in the training and validation cohort

| **Patient characteristics** | **Training (n=146)** | **Validation (n=64)** | ***P*** |
| --- | --- | --- | --- |
| **Age** | 55.80±8.27 | 57.20±9.29 | 0.231 |
| **FIGO stage** |  |  | 0.582 |
| Ⅰ−Ⅱ | 126 (86.3%) | 57 (89.1%) |  |
| Ⅲ−Ⅳ | 20 (13.7%) | 7 (10.9%) |  |
| **Histology** |  |  | 0.692 |
| Endometrioid | 113 (77.4%) | 49 (76.6%) |  |
| Non−Endometrioid | 33 (22.6%) | 15 (23.4%) |  |
| **Type** | n=146 | n=64 | 0.938 |
| Type Ⅰ | 95 (65.1%) | 42 (65.6%) |  |
| Type Ⅱ | 51 (34.9%) | 22 (34.4%) |  |
| **Grade** (only endometrioid, n=162) | n=112 | n=50 | 0.894 |
| G1/2 | 95 (84.8%) | 42 (84%) |  |
| G3 | 17 (15.2%) | 8 (16%) |  |
| **DMI** |  |  | 0.958 |
| - | 109 (74.6%) | 48 (75%) |  |
| + | 37 (25.4%) | 16 (25%) |  |
| **LVSI** |  |  | 0.764 |
| - | 117 (80.1%) | 51 (79.7%) |  |
| + | 29 (19.9%) | 13 (20.3%) |  |
| **LNM** |  |  | 0.739 |
| - | 136 (93.2%) | 59 (92.2%) |  |
| + | 10 (6.8%) | 5 (7.8%) |  |

**FIGO**: the International Federation of Gynecology and Obstetrics; **DMI**: deep myometrial infiltration; **LVSI**: lymphovascular space invasion; **LNM**: lymph node metastasis.

Table S2 The selected radiomic features of the Type, Grade, DMI, LVSI and LNM

| **Features** | **Multivariate logistic regression analysis** | | |
| --- | --- | --- | --- |
|  | **OR** | **95% CI** | ***P*** |
| **Type** |  |  |  |
| firstorder_10Percentile | 46.615 | 5.596-390.225 | <0.001 |
| glcm_JE | 1.981 | 1.349-2.908 | <0.001 |
| wavelet-HLH_firstorder_Skewness | 0.509 | 0.332-0.779 | 0.002 |
| wavelet-LLL_firstorder_10Percentile | 0.09 | 0.012-0.668 | 0.02 |
| glszm_LAE | 1.451 | 1.013-2.080 | 0.043 |
| **Grade** |  |  |  |
| lbp-3D-m2_firstorder_IR | 0.466 | 0.271-0.804 | 0.006 |
| firstorder_Minimum | 2.347 | 1.354-4.161 | 0.003 |
| wavelet-HLH_firstorder_Skewness | 0.305 | 0.141-0.568 | 0.002 |
| glrlm_GLNUN | 2.075 | 1.169-3.682 | 0.013 |
| lbp-3D-k_gldm_DV | 2.705 | 1.358-5.387 | 0.005 |
| wavelet-HLH_glszm_SZNUN | 0.374 | 0.189-0.739 | 0.005 |
| **DMI** |  |  |  |
| wavelet-LLL_firstorder_Mean | 2.339 | 1.518-3.604 | <0.001 |
| lbp-3D-k_glcm_Imc1 | 2.343 | 1.402-3.914 | 0.001 |
| lbp-3D-m2_gldm_DV | 1.870 | 1.243-2.812 | 0.003 |
| wavelet-HLL_firstorder_Median | 2.172 | 1.305-3.616 | 0.003 |
| wavelet-LLH_gldm_LDLGLE | 1.915 | 1.291-2.841 | 0.001 |
| lbp-3D-m2_firstorder_RMAD | 0.543 | 0.348-0.845 | 0.007 |
| **LVSI** |  |  |  |
| wavelet-HLL_glrlm_LGLRE | 2.140 | 1.495-3.063 | <0.001 |
| wavelet-HHH_glszm_SZNU | 1.565 | 1.129-2.169 | 0.027 |
| firstorder_10Percentile | 1.675 | 1.140-2.460 | 0.009 |
| wavelet-LLH_firstorder_Mean | 0.602 | 0.391-0.925 | 0.021 |
| **LNM** |  |  |  |
| wavelet-LLL_firstorder_Kurtosis | 5.020 | 2.070-12.176 | <0.001 |
| glcm_MP | 15.386 | 3.823-61.926 | <0.001 |
| wavelet-HHL_firstorder_TE | 3.545 | 1.707-17.365 | 0.001 |
| gldm_LDE | 0.028 | 0.004-0.209 | <0.001 |
| lbp-3D-k_gldm_HGLE | 2.815 | 1.038-7.634 | 0.042 |
| wavelet-LLH_glrlm_LRE | 6.167 | 1.775-21.462 | 0.004 |
| wavelet-LLL_gldm_GLNU | 0.356 | 0.156-0.814 | 0.014 |
| lbp-3D-k_glszm_ZE | 9.527 | 1.090-83.252 | 0.042 |

**OR**: odds ratio; **CI**: confidence interval; **DMI:** deep myometrial infiltration; **LVSI:** lymphovascular space invasion; **LNM:** lymph node metastasis; **glcm**: gray level co-occurrence matrix; **glszm:** gray-level size zone matrix; **glrlm**: gray level run length matrix; **gldm:** gray level dependence matrix; **JE**: JointEnergy **LAE**: LargeAreaEmphasis; **IR**: InterquartileRange; **GLNUN**: GrayLevelNonUniformityNormalized; **DV**: DependenceVariance; **SZNUN**: SizeZoneNonUniformityNormalized; **LDLGLE**: LargeDependenceLowGrayLevelEmphasis; **RMAD**: RobustMeanAbsoluteDeviation; **LGLRE**: LowGrayLevelRunEmphasis; **SZNU**: SizeZoneNonUniformity; **MP**: MaximumProbability; **TE**: TotalEnergy; **LDE**: LargeDependenceEmphasis; **HGLE**: HighGrayLevelEmphasis; **LRE:** LongRunEmphasis; **GLNU**: GrayLevelNonUniformity; **ZE**: ZoneEntropy

Table S3 ICCs of intra-observer and inter-observer of selected radiomics parameters

| **Features** | **ICC** | |
| --- | --- | --- |
|  | **Intra-observer (95% CI)** | **Inter-observer (95% CI)** |
| **Type** |  |  |
| firstorder_10Percentile | 0.866 (0.346−0.986) | 0.764 (0.209−0.939) |
| glcm_JE | 0.896 (0.604−0.974) | 0.983 (0.719−0.997) |
| wavelet-HLH_firstorder_Skewness | 0.885 (0.546−0.971) | 0.793 (0.375−0.944) |
| wavelet-LLL_firstorder_10Percentile | 0.932 (0.552−0.985) | 0.971 (0.841−0.993) |
| glszm_LAE | 0.998 (0.991−0.999) | 0.974 (0.901−0.993) |
| **Grade** |  |  |
| lbp-3D-m2_firstorder_IR | 0.965 (0.860−0.991) | 0.967 (0.878−0.992) |
| firstorder_Minimum | 0.934 (0.645−0.985) | 0.868 (0.500−0.967) |
| wavelet-HLH_firstorder_Skewness | 0.885 (0.546−0.971) | 0.793 (0.375−0.944) |
| glrlm_GLNUN | 0.806 (0.262−0.951) | 0.913 (0.697−0.977) |
| lbp-3D-k_gldm_DV | 0.939 (0.750−0.995) | 0.848 (0.492−0.962) |
| wavelet-HLH_glszm_SZNUN | 0.967 (0.846−0.992) | 0.936 (0.773−0.984) |
| **DMI** |  |  |
| wavelet-LLL_firstorder_Mean | 0.814 (0.314−0.953) | 0.969 (0.865−0.992) |
| lbp-3D-k_glcm_Imc1 | 0.835 (0.392−0.958) | 0.717 (0.224−0.920) |
| lbp-3D-m2_gldm_DV | 0.918 (0.660−0.980) | 0.848 (0.492−0.960) |
| wavelet-HLL_firstorder_Median | 0.853 (0.366−0.964) | 0.744 (0.224−0.931) |
| wavelet-LLH_gldm_LDLGLE | 0.834 (0.387−0.958) | 0.715 (0.240−0.919) |
| lbp-3D-m2_firstorder_RMAD | 0.981 (0.924−0.995) | 0.954 (0.828−0.988) |
| **LVSI** |  |  |
| wavelet-HLL_glrlm_LGLRE | 0.963 (0.859−0.991) | 0.950 (0.811−0.987) |
| wavelet-HHH_glszm_SZNU | 0.977 (0.909−0.994) | 0.920 (0.725−0.979) |
| firstorder_10Percentile | 0.866 (0.346−0.986) | 0.764 (0.209−0.939) |
| wavelet-LLH_firstorder_Mean | 0.938 (0.734−0.985) | 0.883 (0.579−0.970) |
| **LNM** |  |  |
| wavelet-LLL_firstorder_Kurtosis | 0.862 (0.536−0.964) | 0.926 (0.698−0.982) |
| glcm_MP | 0.780 (0.563−0.946) | 0.862 (0.536−0.964) |
| wavelet-HHL_firstorder_TE | 0.899 (0.608−0.975) | 0.921 (0.725−0.980) |
| gldm_LDE | 0.994 (0.975−0.998) | 0.967 (0.879−0.992) |
| lbp-3D-k_gldm_HGLE | 0.968 (0.872−0.992) | 0.939 (0.773−0.984) |
| wavelet-LLH_glrlm_LRE | 0.985 (0.941−0.996) | 0.819 (0.941−0.996) |
| wavelet-LLL_gldm_GLNU | 0.910 (0.664−0.977) | 0.873 (0.592−0.966) |
| lbp-3D-k_glszm_ZE | 0.836 (0.334−0.959) | 0.907 (0.612−0.977) |

**ICC**: intraclass correlation coefficient; **CI**: confidence interval; **DMI**: deep myometrial infiltration; **LVSI**: lymphovascular space invasion; **LNM:** lymph node metastasis; **glcm**: gray level co-occurrence matrix; **glszm**: gray-level size zone matrix; **glrlm**: gray level run length matrix; **gldm**: gray level dependence matrix; **JE**: JointEnergy **LAE**: LargeAreaEmphasis; **IR**: InterquartileRange; **GLNUN**: GrayLevelNonUniformityNormalized; **DV**: DependenceVariance; **SZNUN**: SizeZoneNonUniformityNormalized; **LDLGLE**: LargeDependenceLowGrayLevelEmphasis; **RMAD**: RobustMeanAbsoluteDeviation; **LGLRE**: LowGrayLevelRunEmphasis; **SZNU**: SizeZoneNonUniformity; **MP**: MaximumProbability; **TE**: TotalEnergy; **LDE**: LargeDependenceEmphasis; **HGLE**: HighGrayLevelEmphasis; **LRE:** LongRunEmphasis; **GLNU**: GrayLevelNonUniformity; **ZE**: ZoneEntropy

Table S4 NRIs of combined model compared with ADC and radiomic model in each group

|  | **NRI** | ***P*** |
| --- | --- | --- |
| **Type** |  |  |
| **Training** |  |  |
| Combined vs. ADC model | 0.358 (0.168−0.548) | <0.01 |
| Combined vs. radiomic model | 0.084 (0.067−0.101) | 0.24 |
| **Validation** |  |  |
| Combined vs. ADC model | 0.286 (0.166−0.406) | 0.02 |
| Combined vs. radiomic model | -0.071 (-0.172−0.03) | 0.18 |
| **Grade** |  |  |
| **Training** |  |  |
| Combined vs. ADC model | 0.179 (0.049−0.309) | 0.03 |
| Combined vs. radiomic model | 0.074 (0.061−0.135) | 0.09 |
| **Validation** |  |  |
| Combined vs. ADC model | 0.310 (0.110−0.510) | <0.01 |
| Combined vs. radiomic model | 0.072 (0.051−0.093) | 0.31 |
| **DMI** |  |  |
| **Training** |  |  |
| Combined vs. ADC model | 0.281 (0.101−0.461) | <0.01 |
| Combined vs. radiomic model | 0.027 (0.011−0.043) | 0.56 |
| **Validation** |  |  |
| Combined vs. ADC model | 0.292 (0.092−0.492) | 0.02 |
| Combined vs. radiomic model | 0.250 (0.119−0.381) | <0.01 |
| **LVSI** |  |  |
| **Training** |  |  |
| Combined vs. ADC model | 0.162 (0.072−0.252) | 0.03 |
| Combined vs. radiomic model | -0.012 (-0.025−0.001) | - |
| **Validation** |  |  |
| Combined vs. ADC model | 0.078 (0.046−0.11) | 0.41 |
| Combined vs. radiomic model | 0.059 (0.032−0.086) | 0.56 |
| **LNM** |  |  |
| **Training** |  |  |
| Combined vs. ADC model | 0.373 (0.123−0.623) | <0.01 |
| Combined vs. radiomic model | -0.015 (-0.155−0.125) | 0.72 |
| **Validation** |  |  |
| Combined vs. ADC model | 0.412 (0.182−0.642) | <0.01 |
| Combined vs. radiomic model | 0.039 (-0.81−0.159) | 0.53 |

**NRI**: net reclassification index; **ADC**: apparent diffusion coefficient; **DMI**: deep myometrial infiltration; **LVSI**: lymphovascular space invasion; **LNM**: lymph node metastasis.

Table S5 Diagnostic Performance of Visual Assessment and Combined Model for DMI and LNM

| **Variables** | **Combined model** | **Reader 3** | **P** | **Reader 4** | ***P*** |
| --- | --- | --- | --- | --- | --- |
| **DMI** |  |  |  |  |  |
| **SEN(%)** | 56.6 | 73.5 | 0.093 | 67.9 | 0.263 |
| **SPE(%)** | 84.1 | 68.7 | 0.001 | 80.0 | 0.006 |
| **ACC(%)** | 77.1 | 80.0 | 0.380 | 71.0 | 0.052 |
| **LVSI** |  |  |  |  |  |
| **SEN(%)** | 93.3 | 53.3 | 0.070 | 73.3 | 0.357 |
| **SPE(%)** | 71.3 | 74.9 | 0.382 | 75.4 | 0.360 |
| **ACC(%)** | 72.9 | 73.3 | 0.313 | 75.2 | 0.665 |

**DMI**: deep myometrial infiltration; **LVSI**: lymphovascular space invasion; **LNM**: lymph node metastasis. **SEN**: sensitivity; **SPE**: specificity; **ACC**: accuracy.
